# Estrogen and viral infection

**DOI:** 10.3389/fimmu.2025.1556728

**Published:** 2025-05-16

**Authors:** Xiangrui Wei, Longyan Hong, Hao Liang, Ke Ren, Wenqing Man, Yinghui Zhao, Pengbo Guo

**Affiliations:** ^1^ Department of Health Inspection and Quarantine, School of Public Health, Cheeloo College of Medicine, Shandong University, Jinan, China; ^2^ Department of Clinical Laboratory Medicine, The First Affiliated Hospital of Shandong First Medical University & Shandong Provincial Qianfoshan Hospital, Shandong Medicine and Health Key Laboratory of Laboratory Medicine, Jinan, Shandong, China; ^3^ Department of Pathogen Biology, School of Clinical and Basic Medicine, Shandong First Medical University and Shandong Academy of Medical Sciences, Jinan, China; ^4^ School of Stomatology, Shandong First Medical University and Shandong Academy of Medical Sciences, Jinan, China

**Keywords:** estrogen, gender difference, immune response, viral infection, molecular mechanism

## Abstract

Gender differences exist in the susceptibility, incidence, progression, and prognosis of diseases caused by viral infections. These differences are influenced by various factors, including lifestyle and habits between males and females. Some reports have pointed out that estrogen plays an important immune-modulatory role in many viral infections. In certain viral infections, estrogen exhibits a protective and regulatory effect, while in others, it has a synergistic and promoting effect. The action of estrogen and its receptors affect the occurrence and prognosis of viral infections to some extent. This article reviews the correlation and the related mechanisms between estrogen and viral infections.

## 1 Introduction

Pathogenic microorganism infections, particularly viral infections, pose a significant threat to human health (1). In recent years, a number of emerging infectious diseases caused by viral infections, including novel coronaviruses, influenza viruses, and others, have emerged, leading to a major public health crisis (2, 3). Although many factors lead to infection, epidemiological data showed that there are gender differences in many pathogenic microorganism infections (4–7). In some viral infection, men showed higher infection susceptibility and worse infection prognosis compared with women. While various factors, such as variations in immune responses and lifestyle habits between genders, may contribute to this phenomenon, growing research suggests that estrogen plays a critical role in viral infections and the associated molecular mechanisms.

This review provides a summary of current research on estrogen-related viral infections and the associated molecular mechanisms.

## 2 Estrogen

Estrogen is a sex steroid hormone that plays a crucial role in the development of female secondary sexual characteristics and the regulation of the reproductive system in adult women. It also demonstrates a wide range of functions and impacts in various aspects (8).Several studies had indicated that virus infection modulated the estrogen expression in host cells (9). Furthermore, estrogen levels impact the host’s susceptibility and immune response to viruses (10). The types of estrogen in the human body include estrone (E1), estradiol (E2), estriol (E3) and estetrol (E4), with estradiol being the most active (8, 11).Estrogen exerts its biological effects primarily by binding to estrogen receptors (ERs). The classical ERs is a nuclear receptor consisting of ERα and ERβ isoforms. Additionally, estrogen can also bind to membrane receptors and exert indirect transcriptional regulatory functions through the second messenger (12).

## 3 Estrogen and virus

### 3.1 Human immunodeficiency virus

HIV, a single-stranded RNA virus belonging to the lentivirus genus of the family Retroviridae (13), causes destruction of the human immune system, particularly CD4+ lymphocytes. This leads to a decrease or loss of immune function and renders the body highly vulnerable to other pathogenic microorganisms. HIV infection is caused by one of two types of retroviruses, HIV-1 and HIV-2. HIV-1 is responsible for the majority of global HIV infections.

Global data suggests that females are more susceptible to HIV-1 infection compared to males, with the reproductive tract mucosa serving as the portal of entry for HIV (14, 15). Estrogen therapy enhances the natural protective barrier of the reproductive tract tissues, effectively preventing the transmission of HIV-1 (16). Mechanisms of estrogen action include increased production of cervical mucus, reduced cervical ectopy, and alteration of vaginal bacterial communities, thereby enhancing anti-HIV infectivity (17).

Studies have found that, compared to males, females exhibit stronger interferon-inducible gene expression in response to HIV-1 infection at the same viral load levels, indicating a more robust immune activation (18). Estrogen plays a crucial role in regulating innate immune function, as evidenced by 17β-estradiol therapy enhancing TLR7-dependent IFNα production in postmenopausal women dendritic cells, thus preventing systemic infection (18). Interestingly, estrogen exhibits protective effects against HIV-1 infection, while progesterone increases susceptibility to HIV-1 (16, 19). Experiments have shown that female macaques exposed to vaginal SIV are less susceptible during the follicular phase of the menstrual cycle (when estrogen levels are higher) compared to the luteal phase (when progesterone levels are higher). During the proliferative phase of the menstrual cycle, elevated progesterone levels typically suppress the immune system, providing opportunities for virus invasion and colonization. Therefore, the high levels of progesterone during the luteal phase may lead to a “susceptibility window” for HIV infection, increasing the risk of vaginal transmission in menstruating women (20, 21). Furthermore, peak estrogen levels downregulate adhesion molecules, reducing the recruitment of inflammatory T cells and macrophages. Progesterone increases the expression of CCR5 on human cervical CD4+ T cells, making females more susceptible (21).

E2 and lactobacilli alleviate the adverse effects of HIV-1 on barrier function of epithelial cells in the reproductive tract and pro-inflammatory cytokine production (22). Estrogen regulates innate immune and inflammatory responses in the female genital tract (FGT), playing a predominant role in reducing local inflammation, and exerting significant anti-inflammatory effects on epithelial cells, such as reducing the secretion of TNF-α, IL-1α, and IL-8 (22, 23). Lactobacilli provide nonspecific defense in the vagina, promoting the immune competence of FGT. Estrogen stimulates the maturation and proliferation of vaginal epithelial cells and accumulation of glycogen, favoring the proliferation of lactobacilli. Lactobacilli produce lactic acid and lower pH to resist HIV invasion (22–24).

Local application of estrogen can also increase the production of keratin proteins in the male foreskin epithelial cells and increase the thickness of the protective layer of epithelial cells, thereby reducing the viral load in contact with Langerhans cells (25, 26), thus contributing to enhanced resistance to HIV infection in males.

HIV also infects astrocytes and microglia, causing neurotoxicity. Infected glial cells can release viral proteins, leading to neuronal damage and resulting in cognitive impairment in patients (13, 27). HIV-1 Tat protein activates HIV-1 LTR-directed gene expression by directly interacting with the transcriptional activation response (TAR) element in the long terminal repeat (LTR) and other cis-acting elements in LTR, thereby promoting HIV-1 infection (28, 29). Studies have shown that physiological levels of E2 and ER-α agonists can inhibit Tat-induced HIV LTR transcription in human astrocytes (27, 30). E2 also regulates neuronal growth by mediating neurite outgrowth and reducing cell apoptosis signals induced by Tat via Erβ (31). Furthermore, exposure to HIV has been shown to decrease the expression of tight junction proteins such as ZO-1, claudin-5, and occludin, thereby disrupting the blood-brain barrier (32). Estrogen can maintain the integrity of the blood-brain barrier by inducing the expression of Sonic hedgehog signaling protein through the activation of the ERα pathway (33, 34).

**Figure 1:**
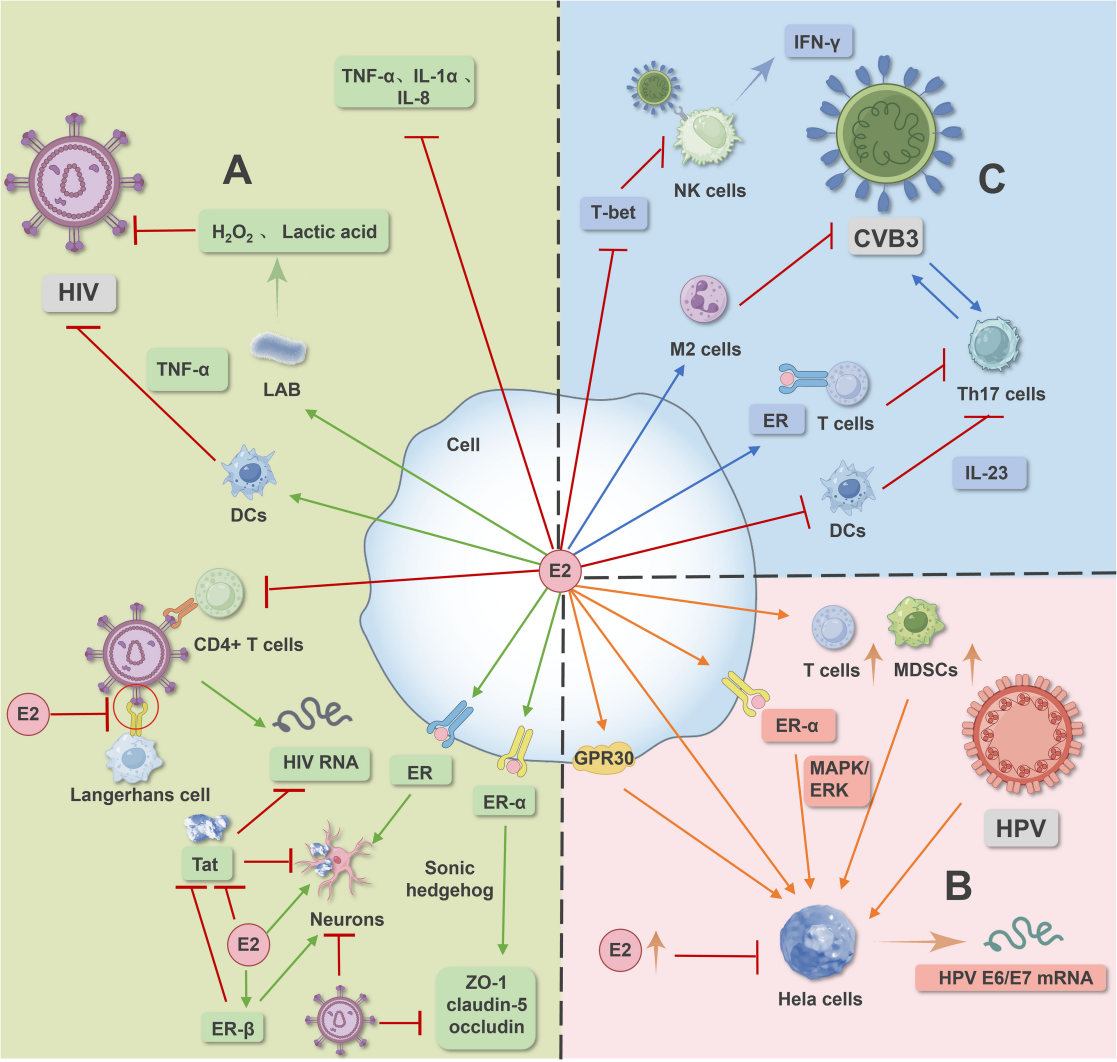
Image 1 illustrates the signaling pathways mediated by estrogen and its receptors in HIV, HPV, and CVB virus infections. In HIV infection **(A)**, estrogen and its receptors modulate signaling pathways to counteract HIV virus-induced infection. In HPV infection **(B)**, estrogen and its receptor-mediated signaling pathways promote human papillomavirus infection and facilitate the occurrence and development of cervical cancer. In CVB virus infection **(C)**, estrogen and its receptor-mediated signaling pathways, along with macrophage polarization and dendritic cell inhibition, regulate CVB infection.

Estrogen can also alter neuronal anti-apoptotic signaling pathways in a receptor-dependent manner to promote neuronal survival, provide nutritional support to neurons and glial cells, promote the expression of nerve growth factor and its receptor, inhibit neuronal inflammation, and exert antioxidant and anti-inflammatory effects (35–37). The mechanism of estrogen action is illustrated in area A of **Figure 1**.

### 3.2 Human papilloma virus

HPV, a DNA virus belonging to the family Papillomaviridae, is associated with various skin infections and is a major etiological factor in cervical cancer. This virus encodes potent oncogenes E6 and E7 (38–40). Despite numerous types of papillomaviruses associated with reproductive tract lesions and malignancies, mere HPV infection alone is insufficient to induce malignant transformation (41, 42).

During HPV infection, estrogen and its receptors play pivotal roles in the occurrence and progression of cervical cancer. Studies indicate that elevated estrogen levels in women with high-risk HPV infections can promote the onset and progression of cervical cancer, with pregnant women diagnosed with cervical cancer having a higher risk of mortality (43–45). Estrogen stimulates HPV16 transcription and increases E6/E7 mRNA levels in cervical cancer cells. Moreover, transfection and immortalization of cells with HPV16 increase 16α-hydroxyestrone, a mechanism through “autocrine” means to prolong estrogen action and enhance cell proliferation. The synergistic effects of HPV and 16α-hydroxylation promote cervical cancer cell proliferation. Progesterone-responsive elements are also observed in the viral promoter region of the HPV16 genome, where progesterone can increase HPV mRNA levels and stimulate viral replication (42, 46–48). The loss of p53, in conjunction with prolonged estrogen exposure and cooperation with HPV oncogenes, promotes the development of cervical and mammary cancers, as well as squamous cell carcinomas in the genital tract (49–51). HPV infection is also recognized as one of the risk factors for head and neck squamous cell carcinoma (HNSCC), with male sex hormones exhibiting potential toxic effects in the development and progression of HNSCC (52).

ERα is essential for the progression and maintenance of cervical cancer in mice, mediating estrogen-stimulated MAPK/ERK signaling pathways, thereby promoting invasion, migration, and proliferation of cervical cancer cell lines. Blocking estrogen receptors can prevent and treat cervical cancer (53–56). Estrogen can also mediate G protein-coupled receptor 30 (GPR30) involvement in the progression of cervical adenocarcinoma (57). GPR30 expression in normal endometrial glands, E2 through GPR30 increases proliferation of cervical columnar and glandular cancer cells, and induces genomic instability exacerbating HPV infection (58). In addition to directly promoting the growth of cervical epithelial cells, estradiol also enhances the function of stromal and infiltrating immunosuppressive cells such as Tregs, MDSCs, and cancer-associated fibroblasts, indirectly promoting tumor growth (45). However, studies have also shown that exogenous high concentrations of estradiol can inhibit the proliferation of HeLa cells and induce apoptosis in HPV-positive cervical cancer cells (45, 59). Dihydrotestosterone can slow down the proliferation of transduced androgen receptor W12 cells and promote the expression of squamous differentiation-related genes, thereby helping to slow the development of cervical epithelial neoplasia (60).The mechanism of estrogen action is illustrated in area B of **Figure 1**.

### 3.3 Coxsackie virus B

CV, known as Coxsackievirus, belongs to the genus enterovirus. It is a non-enveloped, linear, single-stranded RNA virus, which is classified into two subtypes: group A and group B (61). Group A consists of one serotype, while group B consists of six serotypes: CVB1, CVB2, CVB3, CVB4, CV-B5, and CV-B6 (62).

Viral myocarditis is typically caused by viral infections, with one of the most common pathogenic viruses being Coxsackievirus B3 (CVB3), which exhibits gender disparities (63, 64). In males, the incidence and mortality rates of CVB3 infection are higher, possibly due to testosterone enhancement and estrogen suppression, leading to the systemic progression of the disease (65, 66). Following CVB3 infection, male mice generate a virus-specific soluble T-cell response, thereby accelerating disease progression, which may be associated with testosterone levels, while estrogen can protect female mice from exacerbating responses to CVB infection by stimulating antibody production (65).

In males infected with CVB3, T cells play a significant role in the pathogenesis of CVB3-induced myocarditis. More Th17 cells are produced in male mice infected with CVB3, correlating with viral load. Th17 cells adversely affect myocardial cells by promoting virus replication and the production of pro-inflammatory cytokines (TNF-α and IL-1β), exacerbating CVB3-induced myocarditis (66). Compared to testosterone’s negligible effect on Th17 cell induction, estrogen can suppress CVB3-induced Th17 cells by directly interacting with ER-α on T cells or indirectly impairing IL-23 produced by dendritic cells (66, 67).

CVB3 stimulation induces NK cell production of IFN-γ, with male mice being more susceptible to CVB3-induced myocarditis. High doses of estradiol during the menstrual cycle can inhibit NK activity and its mediated cytotoxicity (68). Estrogen affects the production of IFN-γ in NK cells stimulated by CVB3 by downregulating the expression of Th1-specific T-box transcription factor (T-bet), thereby reducing cellular infiltration in male mice and preventing further myocarditis progression (63, 69).

In CVB3-induced myocarditis, there are gender differences in macrophage infiltration phenotypes. M1-type macrophages can produce pro-inflammatory responses and related factors, while M2-type macrophages can produce anti-inflammatory responses and repair damaged tissues (70). Male mice mainly exhibit M1 phenotype macrophage infiltration, while female mice mainly exhibit M2 phenotype macrophage infiltration, and high levels of IFN-γ can also inhibit M2 polarization in male mice (71). Studies have shown that E2 can polarize macrophages towards the M2 phenotype, thereby slowing down the progression of diseases induced by CVB infection (72). Additionally, autophagy can improve cardiac function by limiting myocardial cell death during acute ischemia-reperfusion injury (73–75), with studies indicating that GPR30 can regulate autophagy via the AKT/mTOR signaling pathway to protect myocardial cells (76). The mechanism of estrogen action is illustrated in area C of **Figure 1**.

### 3.4 Hepatitis virus

Hepatitis viruses are the primary pathogens responsible for viral hepatitis. Among them, HAV, HBV, HCV, HDV, and HEV constitute the major global burden of viral hepatitis (77, 78). Research has identified a correlation between estrogen and the infections caused by Hepatitis B virus (HBV), Hepatitis C virus (HCV) and Hepatitis E virus (HEV) (79).

HBV infection leads to hepatocellular degeneration and necrosis, accompanied by varying degrees of inflammatory cell infiltration, which may progress to hepatitis, cirrhosis, and even hepatocellular carcinoma (HCC) (80). Epidemiological investigations indicate that the incidence and prognosis of HBV-related cirrhosis (HBV-LC) and hepatocellular carcinoma (HBV-HCC) are more severe in males and postmenopausal females, possibly due to changes in estrogen levels (79, 81, 82).

HBV affects mitochondrial function to generate reactive oxygen species (ROS) altering the redox state of cells and activate transcription factors to influence virus replication (83). Studies have shown that ER-α can reduce the DNA binding capacity of HNF-4α to the core region of enhancer I, thereby reducing viral load and decreasing the incidence of liver cancer in females infected with HBV (80, 84, 85). Additionally, E2 can protect damaged liver cells by inhibiting the activity of NADH/NADPH oxidase, thereby preventing the secretion of reactive oxygen species (ROS) and TGF-β (81, 86).

Estrogen can enhance the immune activity of NK cells in women infected with HBV through multiple pathways, helping the body clear the virus (87). E2 can also regulate cytokines, attenuate the induction of redox-sensitive transcription factors, and alleviate HBV-related liver damage to slow disease progression (81, 88). The sodium taurocholate cotransporting polypeptide (NTCP) is a functional receptor for HBV infection of liver cells (89). Estrogen can downregulate the expression of NTCP on the surface of liver cells in HBV patients, thereby inhibiting the ability of HBV to infect liver cells (90).

E2 is also considered a protective factor for the progression of fibrosis in patients with chronic hepatitis (91). HBV infection activates hepatic stellate cells, leading to their transformation into myofibroblast-like cells (92). E2 can inhibit the activation of hepatic stellate cells (HSCs) and thus suppress liver fibrosis (81). Chronic HBV infection can cause hepatic steatosis, and the accumulation of hepatic lipids can affect liver fibrosis, inflammation, apoptosis, and cancer (81, 93, 94). Estrogen prevents liver fibrosis caused by hepatic steatosis by altering fat distribution in men to more closely resemble the subcutaneous fat distribution in women (95).

HBV-related HCC is a severe consequence of chronic HBV infection, and estrogen plays an important role in the development of HCC (80). Constitutive activation of the transcription factor STAT3 is observed in the liver tissues and HCC cell lines of nearly half of HCC patients, and it is an important factor inducing cell death (96, 97). Estrogen plays an anticancer or antitumor role by mediating ER-α to inhibit the activation of NF-κB and the release of IL-6, and by attenuating the sustained activity of STAT3 in liver cells (80). In addition, ER-α can inhibit the activity of STAT3 by enhancing protein tyrosine phosphatase receptor O (PTPRO) and the tumor suppressor gene SORBS3α on chromosome 8. The NLR family pyrin domain containing 3 (NLRP3) inflammasome is a critical factor in HCC tissue (98). NLRP3 inflammasome is regulated by the ERβ/mitogen-activated protein kinase (MAPK) pathway, and E2 regulates this pathway to inhibit the proliferation, migration, and colony formation ability of HCC cells (80). Furthermore, estrogen can inhibit the activation of inflammasomes and the transformation activation of tumor-associated macrophages through its receptors, thereby suppressing the growth of HCC (99, 100).

Hepatitis C virus (HCV) is a positive-sense RNA virus that infects the host and leads to the development of hepatitis C. The progression of liver fibrosis and cirrhosis associated with HCV infection is closely related to the development of chronic liver diseases, ultimately culminating in hepatocellular carcinoma (101, 102). Studies have demonstrated that females infected with HCV exhibit higher rates of viral clearance and slower disease progression, indicating a protective role of estrogen in the chronic course of hepatitis C (103, 104). Some protective mechanisms parallel those observed in chronic hepatitis B virus infection: estrogen can inhibit the proliferation and fibrosis of hepatic stellate cells (105).

17β-Estradiol can regulate hepatic cell type I interferon to control HCV infection and mediate ERα inhibition of mature HCV viral particle production (79, 104, 106). Additionally, 17β-estradiol can interfere with the HCV lifecycle through its intracellular receptors, disrupting both early (viral entry) and late (assembly/release) stages of the HCV lifecycle, significantly reducing particle release (107). G protein-coupled estrogen receptor 1 (GPER) is a membrane receptor that specifically binds estrogen and exerts rapid effects on target cells (12). HCV can enter cells via the tight junction protein occludin. Estrogen can bind to GPER, inducing metalloproteinase 9 (MMP) cleavage of tight junction protein occludin to inhibit HCV entry into hepatocytes (108). Estrogen also activates the sterol regulatory element-binding protein (SREBP) pathway, which may interfere with the viral release stage (109). SREBP can regulate microsomal triglyceride transfer protein (MTP), affecting the proper release of infectious particles and regulating apolipoprotein on newly formed particles (110–112).

Research suggests that E2 prevents the accumulation of monocytes and macrophages and suppresses the production of pro-inflammatory cytokines during the course of persistent liver damage caused by HCV through ER. Conversely, progesterone enhances the accumulation of inflammatory response cells and their cytokine production to counteract the positive effects of E2 (113). Testosterone also accelerates the progression of hepatitis C. A cross-sectional study involving male veterans with chronic HCV infection revealed that elevated serum total testosterone increases the risk of advanced HCV infection activity and liver fibrosis (114).

Hepatitis E virus (HEV) is a positive-sense RNA virus belonging to the family Hepeviridae, widely recognized as a major etiological agent of acute viral hepatitis worldwide (115). Infection with HEV can lead to high mortality rates and diffuse hepatic failure, with elevated levels of estrogen during pregnancy associated with increased HEV titers, exacerbating the infection (116, 117). Elevated estrogen levels and its metabolites exert inhibitory effects on hepatocytes, rendering them more susceptible to hepatic dysfunction/failure upon exposure to infectious pathogens (118–120).

In pregnant women infected with HEV, elevated estrogen levels result in placental dysfunction, leading to preterm birth, low birth weight, and fetal death (121, 122). Studies indicate that in HEV infection, estrogen and ERα sustained activation interact with HEV and enhance infection through the cAMPK-PKA-CREB and PI3K-AKT-mTOR signaling pathways, thereby increasing HEV mRNA and protein levels (116, 119, 123).

**Figure 2:**
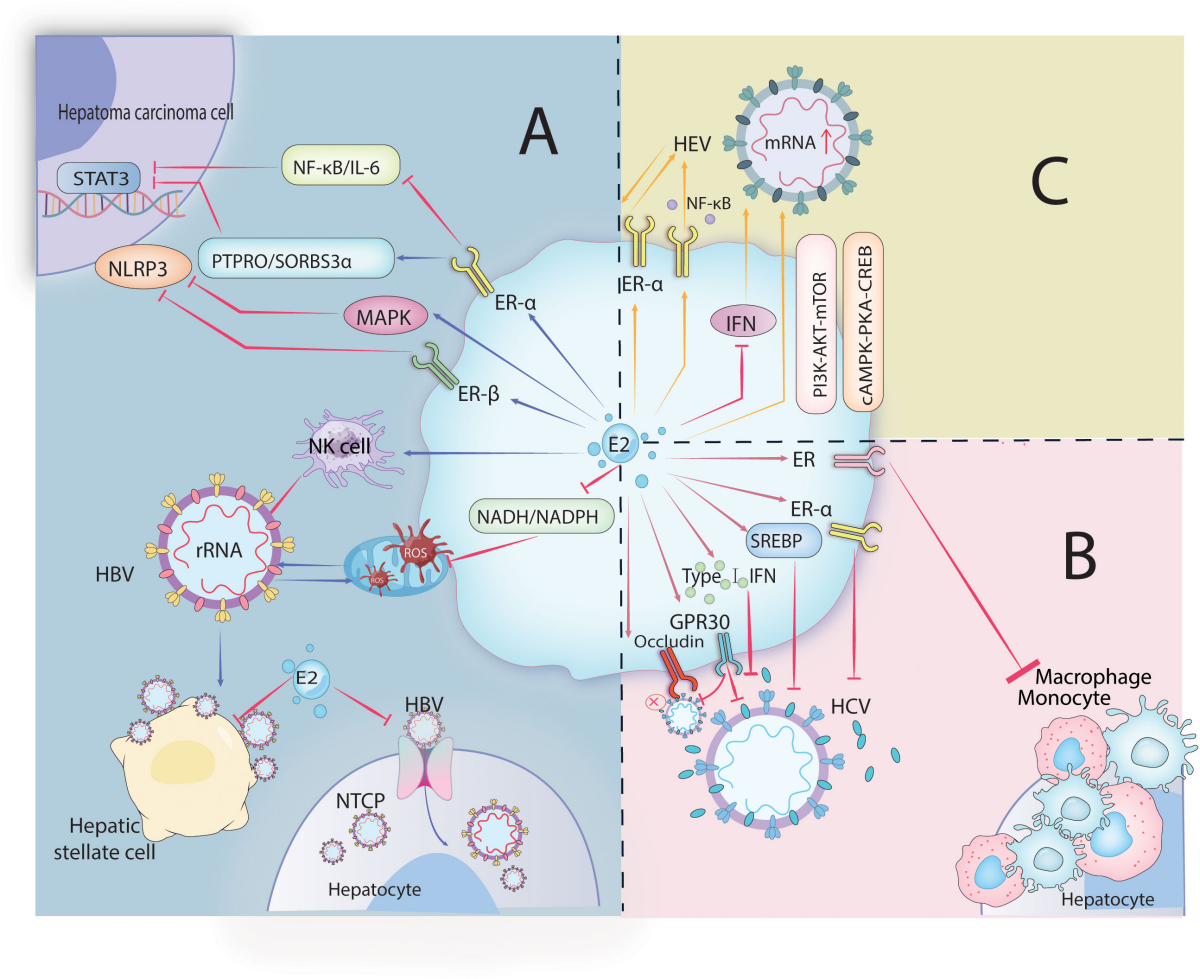
Image 2 depicts the role of estrogen in hepatitis virus infections. HBV, HCV, and HEV. In HBV infection **(A)**, estrogen-mediated receptors inhibit HBV entry into cells and its viral replication, as well as transcription factor activity in hepatoma cells. In HCV infection **(B)**, estrogen and its receptors regulate signaling pathways and interfere with interferon affecting the release of viral particles and inflammatory cell aggregation. In HEV infection **(C)**, estrogen-mediated receptor-regulated signaling pathways promote HEV infection.

Furthermore, estradiol inhibits IFN-mediated antiviral activity within the liver and mediates ERα suppression of the host’s innate immune response during HEV infection to promote HEV infection (117, 122–124). Progesterone also influences the progression of hepatitis E. Pregnant women with fulminant liver failure exhibit elevated levels of progesterone and hepatitis E virus RNA. Studies have reported that progesterone can stimulate ROS production, promote HSCs activation, transforming growth factor expression, and extracellular collagen formation, which may also be critical factors in promoting hepatocellular carcinoma development (125). The mechanism of estrogen action is illustrated in **Figure 2**.

### 3.5 Influenza A virus

Influenza virus is a common respiratory virus belonging to the Orthomyxoviridae family, categorized into four types: A, B, C, and D. Influenza A virus (IAV) is highly pathogenic, causing damage to lung epithelial cells and potentially leading to severe pneumonia, respiratory failure, and acute respiratory distress syndrome (126–130). Female prognosis with IAV infection tends to be worse, often associated with decreased body temperature and significant weight loss (131–134). Despite similar viral titers between genders in lung tissues, females exhibit higher levels of pro-inflammatory cytokines and chemokines, such as TNF-α, IFN-γ, IL-6, and CCL2 (133, 135–137). These inflammatory responses may lead to cytokine storms, significantly increasing morbidity and mortality associated with IAV infection (138–142).

IAV infection also impairs ovarian function and decreases estrogen and progesterone levels in female mice, with pregnancy being a risk factor for severe outcomes related to influenza (132, 143). The role of estrogen in IAV infection is mediated through pro-inflammatory responses, where low concentrations of estradiol promote excessive inflammation and disease progression (144). However, high-dose estradiol exerts anti-inflammatory effects by inhibiting NF-kB transcriptional activity and mediating the ERα signaling pathway, suppressing the transcription of pro-inflammatory genes and cytokine production, thereby reducing the production of lung TNF-α and CCL2 and improving survival rates (145).

Physiological concentrations of progesterone can also promote faster repair of damaged lung tissues to prevent fatal and sublethal IAV infections (132). The mechanism primarily involves progesterone promoting higher concentrations of anti-inflammatory cytokines and lower concentrations of pro-inflammatory cytokines, reducing nitric oxide production, while maintaining macrophages and dendritic cells in a less activated state, thereby exerting protective effects during IAV infection (146–149).

Studies have shown that in lung tissues infected with the influenza virus, males exhibit faster resolution of inflammation and tissue repair compared to females, possibly due to elevated levels of certain growth factor proteins and testosterone levels in males (150). Testosterone can promote the contraction of pneumonia inflammatory monocytes during the peak of the disease and control the frequency of lung-specific CD8+ T cells and eosinophils, thereby improving the outcome of IAV infection (151).

### 3.6 Severe acute respiratory syndrome coronavirus 2

SARS-CoV-2, a member of the Coronaviridae family, is an RNA virus with high transmissibility and pathogenicity. It emerged in late 2019, precipitating the global COVID-19 pandemic. COVID-19 manifests as pulmonary inflammation and acute respiratory distress syndrome, posing significant threats to human health and public safety (152, 153). Epidemiological data indicate gender disparities in the incidence and mortality rates of COVID-19, with males exhibiting higher rates of infection, mortality, and disease severity compared to females (154). Studies suggest that estrogen and its receptors contribute to this gender difference, exerting effects through various mechanisms such as modulating the cellular entry of SARS-CoV-2, the immune response, and influencing coagulation function (155).

Angiotensin-converting enzyme 2 (ACE2) is expressed in various epithelial cells, including those in the lungs and respiratory tract, where it regulates fluid balance and the renin-angiotensin-aldosterone system (RAAS) (154, 156). SARS-CoV-2 can bind to ACE2 to enter cells, concurrently downregulating ACE2 expression, leading to an imbalance between ACE2 and Ang II levels, affecting the human RAAS system (157). Additionally, the spike protein of SARS-CoV-2 interacts with ACE2, facilitating membrane fusion and releasing viral RNA by binding to ACE2 on the host cell surface, thereby accelerating the progression of respiratory diseases (154, 158). Estrogen-responsive elements are present in the promoter region of ACE2, exerting their effects by regulating ACE2 gene expression, resulting in lower ACE2 expression levels in the lungs of females compared to males, thereby reducing interactions with the viral spike protein (159, 160). ACE2 glycosylation can enhance the stability and affinity of virus spike protein binding to host cell receptors. Estrogen, by binding to almost all sites including ACE2 glycan, inhibits host viral infection, reducing receptor surface energy, making it less prone to interact with the virus (161).

Estrogen also exerts anti-inflammatory effects by modulating innate and adaptive immunity, thereby inhibiting COVID-19 progression. SARS-CoV-2 infection leads to increased levels of Th1 cytokines interferons, inflammatory cytokines, and monocyte chemoattractant protein-1 (MCP-1) (162, 163). These cytokine storms activate monocytes/macrophages and neutrophils, leading to multi-organ failure, acute respiratory distress syndrome, and disseminated intravascular coagulation (158). Studies indicate that estrogen therapy attenuates MCP-1 expression in the blood, alleviates endothelial dysfunction, thereby inhibiting the occurrence of inflammatory responses and improving trauma outcomes (164). Infected lung epithelial cells produce IL-6, IL-1β, and IFN-I/III, which induce inflammatory programs in macrophages and recruit inflammatory monocytes, granulocytes, and lymphocytes from circulating blood, causing inflammatory responses (165). Estrogen receptors (ERs) are transcription factors crucial in regulating immune cell development and both innate and adaptive immune pathways (166, 167). High concentrations of estrogen upregulate ER signaling, suppress cytokine storms, and clear accumulated inflammatory cells (168).

*In vitro* experiments suggest that estrogen therapy can also regulate the NF-κB signaling pathway and the production of IL-6 and TNF-α (164, 166). E2 downregulates the expression of monocyte chemotactic factor receptors 2 (CCR2) and 3 (CXCR3) in mice, reducing monocyte recruitment (169). Moreover, estrogen inhibits the production of pro-inflammatory cytokines (including IL-6, IL-1β, and TNF-α) by monocytes and macrophages, exerting innate immune anti-inflammatory effects, while also preventing the migration of neutrophils and monocytes, among other innate immune cells, to the inflammatory area, thereby slowing disease progression (153).

Most severe COVID-19 patients exhibit reduced T lymphocytes. E2 promotes extrathymic T cell differentiation, affecting thymic tissue cell growth, and plays a role in immune system regulation. Specifically, estrogen alters the number and function of peripheral blood T lymphocytes through the sex hormone receptor pathway and conveys relevant information to the immune system (170). Maintenance of immune function typically relies on moderate lymphocyte apoptosis and Th1/Th2 balance, where Th1 and Th2 mutually inhibit each other (171). COVID-19 pathogenesis is complex, but characterized by Th1/Th17 immune responses. Overactivation of Th1/Th17 cells leads to the release of pro-inflammatory cytokines, resulting in lung injury (172). Th2-type immunity and regulatory T cells (Tregs) have the ability to alleviate systemic inflammation and complications. Estrogen therapy reduces the secretion of Th1 cytokine IFN-γ and promotes the secretion of Th2 cytokine IL-4, thereby correcting Th1/Th2 imbalance and enhancing the body’s ability to combat extracellular microbial infections (173). Tregs limit immune pathological reactions in the lungs of respiratory virus-infected patients. Tregs are significantly reduced in COVID-19 patients, estrogen promoting Treg cell expansion playing a protective role in SARS-CoV-2 infection (174, 175).

In terms of cellular immunity, estrogen stimulates plasma cells to produce immunoglobulins, regulates B cell maturation, upregulates the expression of pro-B cell survival mediators, and downregulates the expression of pro-B cell apoptosis mediators, thereby slowing SARS-CoV-2 infection (176). Estrogen also regulates the number of eosinophils and increases their migration, adhesion, survival, and degranulation *in vivo* and *in vitro*, affecting adaptive immunity (169, 177). Eosinophils can produce antiviral molecules and participate in the adaptive immune response caused by SARS-CoV-2 (178).

Due to the highly inflammatory response, platelet activation, endothelial dysfunction, and congestion, COVID-19 patients are prone to thrombotic diseases (179). Estrogen activates endothelial nitric oxide synthase transcription by binding to classical ER or GPR30, upregulating nitric oxide expression, maintaining endothelial homeostasis, and vasodilation (180). *In vitro* experiments have also shown that estrogen inhibits platelet aggregation by promoting the efflux or reuptake activity of Ca2+ (181).

SARS-CoV-2 infection is related not only to estrogen but also to testosterone and progesterone. High levels of testosterone promote virus entry, adversely affecting male resistance to COVID-19 (182). Transmembrane serine protease 2 (TMPRSS2) can cleave ACE2 and promote virus entry into host cells (183, 184). Androgen receptor activity is necessary for TMPRSS2 gene transcription, with high levels of testosterone upregulating its expression, making males more susceptible to COVID-19 (185). Unlike testosterone, progesterone has a protective effect in SARS-CoV-2 infection. Progesterone can promote CD4+ T cell bias from Th1 to Th2, promote anti-inflammatory responses, and regulate Siglec receptor to exert direct antiviral effects (186, 187). The mechanism of estrogen action is illustrated in area B of supplementary figure.

## 4 Conclusion

Gender-based disparities in disease incidence and mortality following viral infections have been extensively documented, with variations in hormonal profiles between males and females and the differential involvement of hormones in response mechanisms (7, 188). Hormones also exhibit synergistic and antagonistic interactions. Evidence suggests estrogen’s regulatory role in human susceptibility to viruses and immune responses (189, 190). Disparities in estrogen levels between genders and the pathways mediated by hormones may be pivotal factors in the divergent outcomes post-viral infection. As previously mentioned, males demonstrate heightened susceptibility, severe illness, and poorer prognoses across most viral infections. Estrogen exerts protective effects post-infection, attenuating disease onset and progression. However, recent studies have unveiled estrogen’s synergistic role with human papillomavirus (HPV) in promoting cervical carcinogenesis. Furthermore, elevated estrogen levels and ERα activation during pregnancy facilitate hepatitis E virus (HEV) infection in females. The protective effects of estrogen are typically realized through immunomodulation and anti-inflammatory actions (145, 189). Estrogen also influences macrophage classification, promoting an increase in M2 macrophage numbers, thereby aiding in disease control (191). Estrogen typically necessitates ER binding to modulate the estrogen receptor signaling pathway, thereby further regulating other signaling pathways. Apart from its anti-inflammatory effects, estrogen exhibits varied protective effects against different viral infections.

Numerous clinical studies have substantiated the therapeutic potential of exogenous estrogen therapy. Exogenous estrogen or ERα agonist therapy may be necessary for diseases where estrogen confers beneficial effects in viral infections (192–194). Conversely, ERα antagonists may hold therapeutic benefits for HPV, HEV, and other viruses. Future research should delve into elucidating the specific biological mechanisms of estrogen in viral infections, exploring improved estrogen therapy methods, including dosage, administration routes, and more appropriate targets, while balancing these aspects with other health risks.

## Supplementary material

Supplementary Figure 1 The role of estrogen in influenza virus and SARS-CoV-2 infections. In influenza virus A (IVA) infection **(A)**, low concentrations of estrogen promote the release of inflammatory factors, while high concentrations of estrogen, mediated by its receptors, inhibit the release of inflammatory factors through signaling pathways. In SARS-CoV-2 infection **(B)**, estrogen and its receptor regulate signaling pathways and immune cells and proteins to counteract viral infection.
