# Supplementary figures and images for: Estrogen and viral infection
Source: Front Immunol. 2025 May 16;16:1556728. doi: 10.3389/fimmu.2025.1556728 (PMC12122754; doi:10.3389/fimmu.2025.1556728)

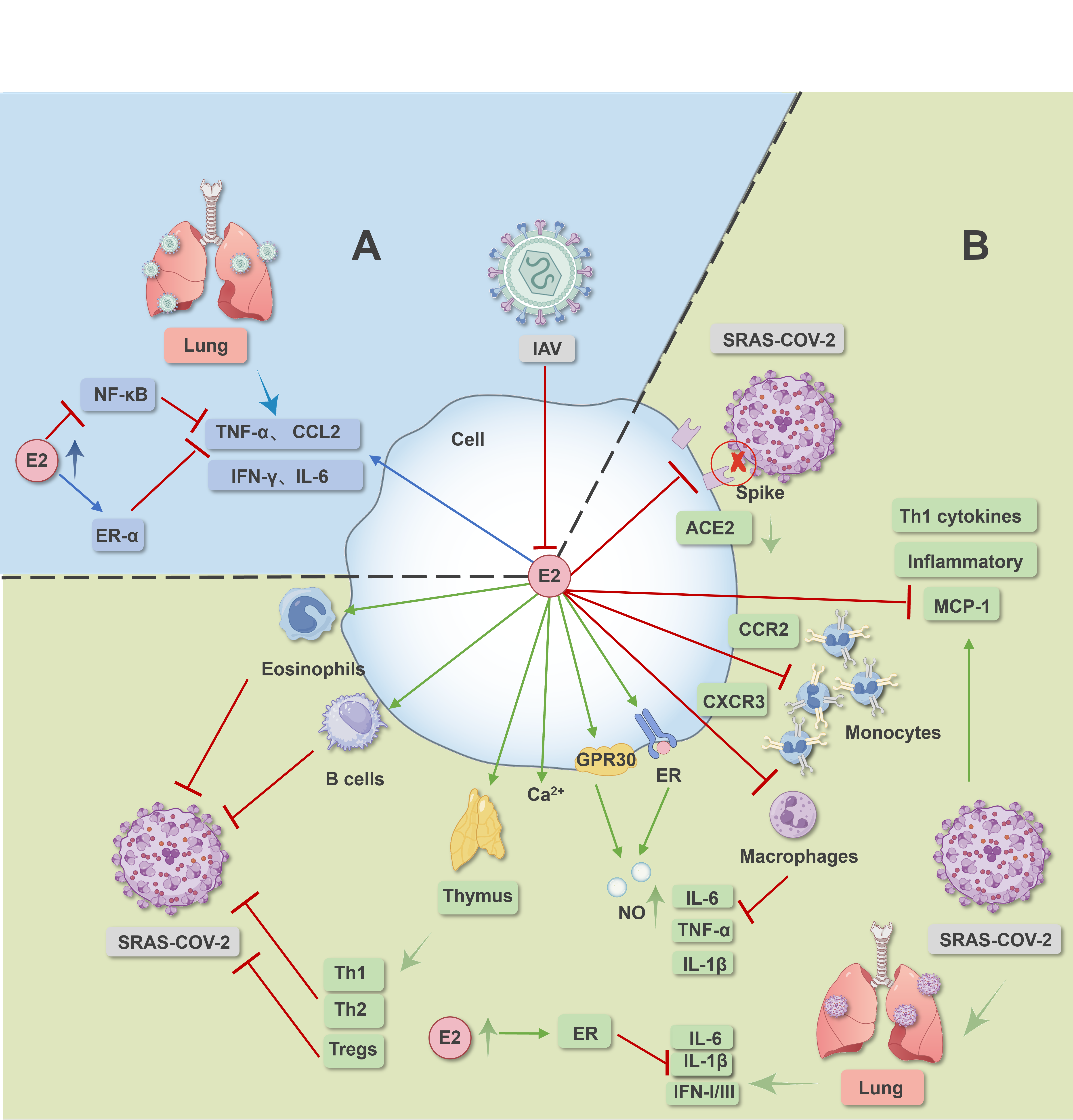

Supplement: Supplementary Figure 1 — The role of estrogen in influenza virus and SARS-CoV-2 infections. In influenza virus A (IVA) infection (A), low concentrations of estrogen promote the release of inflammatory factors, while high concentrations of estrogen, mediated by its receptors, inhibit the release of inflammatory factors through signaling pathways. In SARS-CoV-2 infection (B), estrogen and its receptor regulate signaling pathways and immune cells and proteins to counteract viral infection. [file Image1.tif]
